# An Extended Photoperiod Increases Milk Yield and Decreases Ovulatory Activity in Dairy Goats

**DOI:** 10.3390/ani10101879

**Published:** 2020-10-15

**Authors:** Kathryn J. Logan, Brian J. Leury, Vicki M. Russo, A.W.N. (Sandy) Cameron, Alan J. Tilbrook, Frank R. Dunshea

**Affiliations:** 1 Faculty of Veterinary and Agricultural Sciences, The University of Melbourne, Parkville 3010, Victoria, Australia; kath612@yahoo.com.au (K.J.L.); brianjl@unimelb.edu.au (B.J.L.); victoria.russo@agriculture.vic.gov.au (V.M.R.); 2 Agriculture Victoria, Ellinbank 3820, Victoria, Australia; 3 Meredith Dairy, 106 Camerons Road, Meredith 3333, Victoria, Australia; sandy@meredithdairy.com; 4 Centre for Animal Science, Queensland Alliance for Agriculture and Food Innovation, The University of Queensland, St Lucia 4067, Queensland, Australia; a.tilbrook@uq.edu.au; 5 Faculty of Biological Sciences, The University of Leeds, Leeds LS2 9JT, UK

**Keywords:** lactation, goat, buck effect, photoperiod, progesterone, ovulation

## Abstract

**Simple Summary:**

Short day length is associated with reduced milk yield in dairy ruminants possibly as animals prepare for the subsequent reproductive cycle. This study was conducted to determine the effect of an artificially increased daylength on milk production and ovulation in lactating goats. Increased daylength increased milk yield although the responses were only apparent during late lactation. Increased daylength reduced ovulation rate as lactation advanced although this reduction could be partially mitigated by exposing the lactating females to entire males. These findings suggest that artificially extending daylength can increase milk production and persistence while decreasing ovulatory activity in dairy goats.

**Abstract:**

Short day length is associated with reduced milk production in dairy ruminants. Dairy ruminants have been kept in lit sheds during winter to extend the day length and stimulate milk production. However, there studies are few on the effect of an extended photoperiod on the ensuing reproductive performance of dairy goats. The aim of this study was to examine the effect of long day photoperiod (LDPP) and exposure to bucks on milk production and plasma progesterone and prolactin in dairy goats. The study was conducted in 122 non-pregnant lactating dairy goats over 18 weeks from April to August (late autumn and winter in the Southern Hemisphere). The goats were kept in open sided sheds in which the control treatment received ambient lighting while the LDPP treatment received 16 h of light, including artificial lighting. In June, July and August synchronised does were randomly assigned each month to the presence or absence of a buck and ovulatory activity determined from plasma progesterone. Plasma progesterone concentrations were reduced (0.73 vs. 0.46 pmol, *p* < 0.001) while prolactin concentrations were increased (0.095 vs. 1.33 ng/mL, *p* < 0.001) in LDPP goats. The former response was most marked in late winter (0.58 vs. 0.004 pmol, *p* < 0.001) indicating a lack of functional corpora lutea. While there was no overall effect of buck exposure on plasma progesterone concentrations there was a three-way interaction such that plasma progesterone concentrations were increased (*p* < 0.05) by exposure to bucks in LDPP goats in August (late winter) but not at other times. Milk production was increased in LDPP goats over the latter stages of the study (1. 55 vs. 1.82 L/d, *p* < 0.05). Also, persistency of lactation was greater in LDPP goats with fewer goats drying off (13 vs. 0%, *p* < 0.05). These findings suggest that LDPP can increase milk production and persistence while decreasing ovulatory activity in dairy goats.

## 1. Introduction

The seasonal reproductive activity of temperate and subtropical goats is largely influenced by photoperiod [1,2]. As the days begin to shorten in Autumn, a regular oestrous cycle is initiated and when days are lengthening in Spring, anoestrus occurs. These responses to changing photoperiod have been utilised commercially by exposing goats to 16 h of light during winter through the provision of supplemental lighting. The goats are then returned to ambient shorter day lengths, which results in mating activity in spring. In this production system, the goats are six months out of phase with when they would naturally cycle, enabling year-round milk production if implemented across some of the herd. However, there is little scientific literature to support this practice of delaying ovulation. 

The artificial extension of day length has also stimulated milk production in several dairy ruminants, including goats [3,4,5,6]. The studies by Garcia-Hernandez et al. [3] and Russo et al. [6] indicate that the response in milk production to extended light exposure may vary with both time of year and stage of lactation with responses being greater after peak lactation and when daylength is declining. Then again, goats may eventually become refractory to the stimulatory effects of prolonged day length if the treatment period is too long [5]. The application of an extended photoperiod on lactating dairy goats may provide a practical, non-invasive method to substantially increase their milk yield while delaying ovulation to allow for year-round milk production. 

The natural breeding season of the goat doe can be brought forward by the presence of a buck, this phenomenon is known as the buck effect. The isolation of does from bucks for a period, at a distance that limits sight, sound, odour and touch can induce ovulation in anovulatory does when bucks are reintroduced [7]. The buck effect is a result of pheromones that are produced in the sebaceous gland of the skin [8]. The presence of a buck stimulates an olfactory cue, inducing ovulatory activity in a high proportion of does within 3 days [9]. The effect of buck exposure on lactating does that have been exposed to an extended photoperiod are unknown, but it is hypothesised that buck exposure will increase ovulatory activity in does exposed both to natural or extended photoperiods during Autumn and Winter.

Therefore, it is proposed that artificially extending day length (to 16 h day length) would increase milk production and delay ovulation in dairy goats and that buck exposure will increase ovulation.

## 2. Materials and Methods

### 2.1. Animals and Feeding

The study involved total of 122 Saanan and Saanan British Alpine cross dairy goats from Meredith Dairy Victoria, Australia (Latitude 37°51′ OS). The does ranged in age from two to seven years and had all kidded prior to the experiment in late November/early December 2009. Kids were weaned within 24 h or birth. Does were stratified according to milk production in the week prior to commencing the experiment with only does producing more than 1.5 L/d being included in the study. 

The animals were housed within their treatment groups, in open sided sheds with fresh bedding applied daily, with 1.5 square metres of floor space per animal provided. Feed was provided *ad libitum* as a total mixed ration consisting of cereal hay (333 g/kg), vetch hay (167 g/kg), barley (233 g/kg), oats (133 g/kg), canola meal (100 g/kg) and molasses (3 g/kg), calculated to provide 32% neutral detergent fiber, 14% crude protein and 10 MJ ME per kg dry matter. A commercial mineral mix was added, along with 15 g sodium bicarbonate and 15 g bentonite.

The experiment was approved by The University of Melbourne Faculty of Science, School of Land & Environment, and Optometry & Vision Sciences Animal Ethics Committee (Protocol number 0911209.1).

### 2.2. Experimental Design

Animals were randomly allocated to either the control (*n* = 61) or the long day photoperiod (LDPP) (*n* = 61) group. The control group received natural lighting and the LDPP group received 16 h of light, a combination of natural and artificial lighting. Treatment groups were housed in separate but co-located sheds to ensure there was no inadvertent exposure of control goats to artificial lighting. The treatments were applied for eighteen weeks beginning on 8 April (mid-autumn) (129 ± 2.7 days in milk) and finishing 13 August 2010 (late winter). Before commencing the study, each group was housed in their allocated sheds for one week as an acclimatisation period, during which time both groups received a natural lighting regime. Artificial supplemental lighting was provided by overhead fluorescent lights, designed to provide 200 lux at eye level. The lights came on at 1700 h and turned off at 2330 h each day, as sunrise during this period was at approximately 0730 h and sunset at approximately 1730 h. During the experimental period the ambient day light fluctuated from 11 h 40 min on 1 April, decreasing to 9 h 33 min on 21 June before increasing to 10 h 35 min on 13 August.

In order to determine the reproductive status of the goats their reproductive cycles were synchronised using a progesterone/prostaglandin protocol. All experimental does had an Eazi-Breed Controlled Internal Drug Release Sheep & Goat device (CIDR) inserted intravaginally for a period of 9 days each month prior to removal on the 1 June, 30 June and 2 August. At CIDR removal goats were injected with 0.5ml Prostaglandin analogue (Estrumate: Schering-Plough Animal Health Limited, 11 Gibbon Rd, Baulkham Hills, NSW 2153, Australia) intramuscularly.

In the months of June, July and August goats within each photoperiod treatment were randomly allocated to either the presence or absence of a buck treatment (*n* = 27–30 for each photoperiod x buck x month group). Does that were not exposed to a buck remained within the milking herd whilst does exposed to a buck were isolated under the appropriate light treatment with bucks for 48 h. The ratio of bucks to does was 1 to 5. The does continued to be milked during buck exposure. Oestrous behaviour was monitored during buck exposure and any does that displayed oestrus were returned to the milking herd to prevent bucks concentrating on individual does. After each buck exposure period the remaining does were returned to the appropriate light treatments within the milking herd. Each month the goats were re-randomised prior to allocation to buck treatment.

### 2.3. Measurements

The does were milked twice daily at 0600 and 1600 h, in a 36-sided herringbone system fitted with automatic cup removers and in-line electronic milk meters. The milking sheds were fitted with MM25SG milk meters (DeLaval), and the data were managed with an ALPRO processor and accessed through ALPRO, a Windows based program provided by De Laval. Milk volume was recorded daily and the average for the entire week was taken. Live weights of all experimental animals were recorded on the days when blood samples were collected and when animals were sorted into buck treatment groups. The animals were weighed on an electronic sheep/goat weight crate with automatic recording. The weighing crate was manufactured by Pratley (Temuka, New Zealand). Eartags were read with an Allflex antenna (Palmerston North, New Zealand) and data captured using a Tru-test XR 3000 reader (Auckland, New Zealand) at half kilogram increments.

Blood samples (10 mL) were collected 11 days after synchronisation ended at CIDR removal from all goats via jugular venipuncture, using evacuated tubes containing lithium heparin. This sampling regime was chosen as preliminary research had shown that plasma progesterone concentrations above 2 ng/mL at 11 days after CIDR removal could be used as proxy for ovulation in goats from this herd [10]. Once blood had been collected, the goats were injected intramuscularly with Estrumate to avoid conception and to permit continued cyclic activity. Blood samples were refrigerated until centrifugation at 3000 rpm for 10 min. Plasma was harvested and stored at −20 °C until progesterone and prolactin analyses.

### 2.4. Plasma Hormone Analysis

The plasma concentration of progesterone was assayed using an extracted radioimmunoassay which was developed for sheep plasma. The extraction involved vortexing the samples with hexane and freezing the aqueous layer, then decanting the supernatant and drying it down under air. This was then reconstituted in buffer and assayed. The extraction step is necessary to separate the progesterone from binding proteins. In this assay the standards are also extracted (they have charcoal stripped plasma added to them). Progesterone (P0130, Sigma Aldrich, St. Louis, MO, USA) was used as standard and [1,2,6,6-3H]Progesterone (NET381, Perkin Elmer, Melbourne, Australia) as the tracer. The progesterone antiserum S23 was raised in sheep against progesterone-11-a-BSA (supplied by Dr J Malecki, Bairnsdale, VIC). The progesterone assays were conducted with a sensitivity of 0.300 to 0.386 pmol/mL. The plasma prolactin concentrations were assayed in duplicate using Sigma (St. Louis, Mo, USA), NIDDK-oPrl-I-1 as standard and 125I mono iodinated ovine prolactin as a tracer. The prolactin antiserum NIDDK-anti-oPrl-2 was raised in sheep. The prolactin assays were conducted with a sensitivity of 0.103 to 0.171 ng/mL. 

### 2.5. Statistical Analysis

The effects of photoperiod treatment and week of treatment, as well as the interaction between them on milk yield, were all evaluated using restricted maximum likelihood (REML) suitable for a repeated measure analysis with individual goat and parity as random effects for milk yield (Genstat Version 16, VSN International Ltd., Hemel Hempstead, UK). Plasma hormone data were analysed for the main and interactive effects of photoperiod, month of treatment and buck exposure with individual goat and parity as random effects. Some data that exhibited heterogeneity in the variance (e.g., plasma prolactin) were log-transformed before analysis. The proportion of goats that displayed oestrus or became dry were analysed by chi-square (χ^2^) goodness of fit. 

## 3. Results

**Figure 1:**
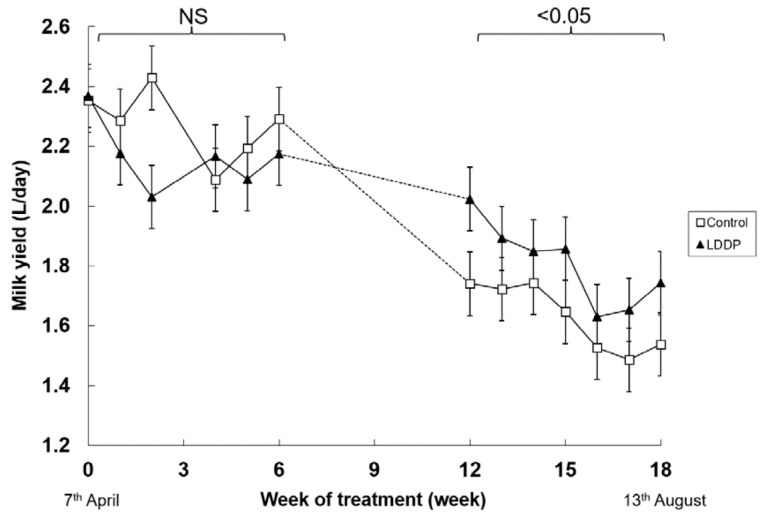
Effect of ambient lighting (Control) or long day photoperiod (LDPP) on milk yield in lactating goats in winter in the Southern Hemisphere. Data are means ± standard error of the difference for the interaction between treatment and week. NS: no significant.

Unfortunately, there was a malfunction with the milk recording system between weeks 6 and 12 and so there are no reliable milk yield data for this period. Nevertheless, the rest of the data were able to be analysed for the effects of photoperiod and week of treatment. There was no significant main or interactive effects of photoperiod on milk yield during the first 6 weeks of treatment (Figure 1). Milk yield declined between weeks 12 and 18 of treatment as lactation advanced, particularly in the goats that were not exposed to the LDPP. As a consequence, milk yield was higher in the LDPP group over this period (1.55 vs. 1.82 kg/d, *p* < 0.05). Also, the proportion of goats that dried off during this period was lower in goats that had been exposed to a LDPP (13 vs. 0%, *p* < 0.01). There were no significant differences in liveweight in response to LDPP (73.7 vs. 71.8 kg, *p* < 0.14) nor any interactions between photoperiod and week of treatment (*p* < 0.13) (data not shown).

**Figure 2:**
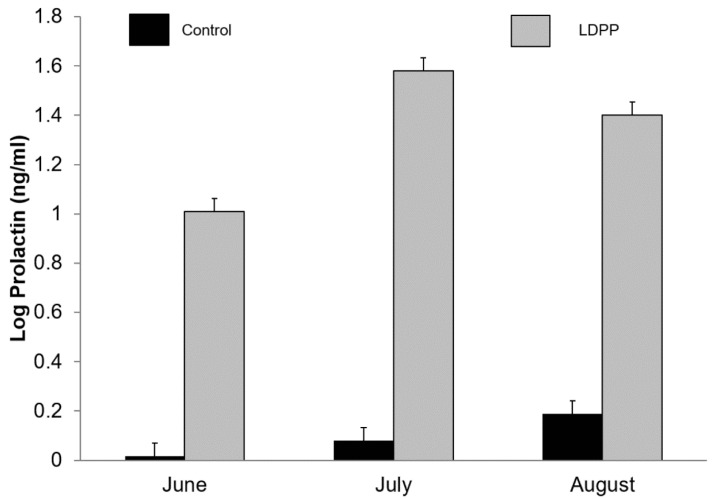
Effect of ambient lighting (Control) or long day photoperiod (LDPP) on plasma prolactin (log-transformed) in lactating goats in winter in the Southern Hemisphere. Data are means ± standard error of the difference for the interaction between treatment and month.

Plasma prolactin increased as lactation advanced (*p* < 0.001) and was higher in goats exposed to LDPP (1.10 vs. 21.5 ng/mL, *p* < 0.001; Figure 2). There was an interaction (*p* < 0.001) between month and photoperiod such that plasma prolactin progressively increased over time in those goats not exposed to LDPP whereas for those exposed to LDPP plasma prolactin peaked in July (Figure 2). There were no main or interactive effects of buck exposure on plasma prolactin concentrations (data not shown). 

**Figure 3:**
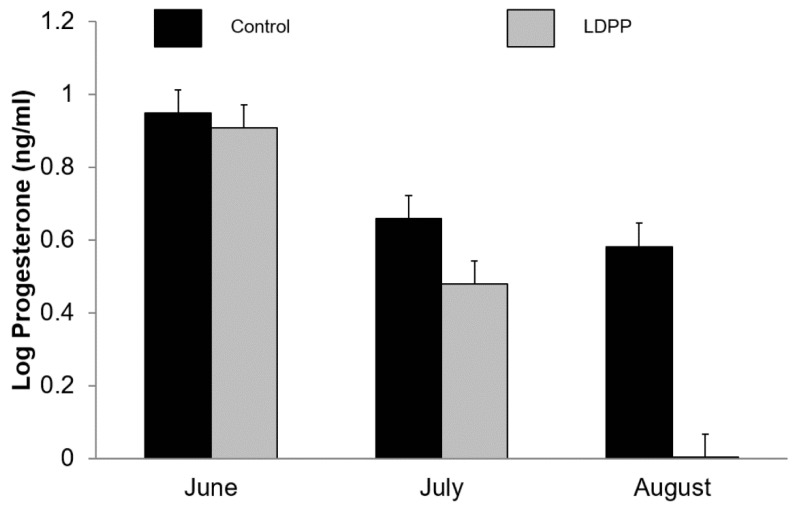
Effect of ambient lighting (Control) or long day photoperiod (LDPP) on plasma progesterone (log-transformed) in lactating goats in winter in the Southern Hemisphere. Data are means ± standard error of the difference for the interaction between treatment and month.

**Figure 4:**
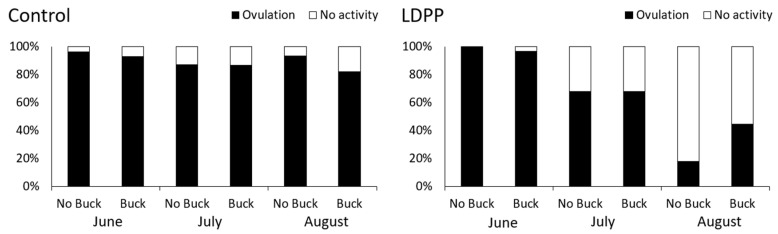
Effect of ambient lighting (Control) or long day photoperiod (LDPP) and buck exposure on ovulatory activity in lactating goats in winter in the Southern Hemisphere (*n* = 27–30 for each photoperiod × buck × month group).

Plasma progesterone decreased as lactation advanced (*p* < 0.001) and was lower in goats exposed to LDPP (5.35 vs. 2.90 ng/mL, *p* < 0.001; Figure 3). There was an interaction (*p* < 0.001) between month and photoperiod such that plasma progesterone concentrations decreased to a greater extent over time in those goats exposed to LDPP (Figure 3). While there was no main effect of buck exposure on plasma progesterone concentrations (*p* = 0.23) there was a three-way interaction (*p* = 0.034) between buck exposure, light treatment and month such that buck exposure increased plasma progesterone in does exposed to LDPP in the third month of treatment (data not shown) and this is reflected in the ovulation rates (Figure 4). 

There was no significant difference between the number of goats that ovulated in either buck exposed group or in the no buck experimental group during June (*x*^2^ = 0.315, *p* = 0.57), July (*x*^2^ = 0.0025, *p* = 0.96) or August (*x*^2^ = 1.71, *p* = 0.19) of the goats treated with natural light (Figure 4). For those goats exposed to LDPP there was no significant difference between the number of goats that ovulated in the buck or no buck exposed group during June (*x*^2^ = 1.05, *p* = 0.31) and July (*x*^2^ = 0.00, *p* = 1.00). However, during August the number of does exposed to LDPP that ovulated in response to the buck exposure group was higher than the number ovulating in the goats that were not exposed to bucks (*x*^2^ = 4.55, *p* = 0.033) (Figure 4). 

## 4. Discussion

The major finding from the present study was that a LDPP can increase milk yield and persistence while decreasing ovulatory activity in lactating dairy goats. Also, the decrease in ovulatory activity as a result of LDPP can be partly ameliorated by exposure to bucks. The LDPP had a strong influence on the endocrine status of the goats with a marked increase in plasma prolactin and decrease in plasma progesterone concentrations.

These findings of the current study are consistent with those of others who found milk yield was increased by LDDP in goats [3,6,11]. In the present study there was an effect on milk yield over the latter stages of the study whereas there was no effect in the first 6 weeks of exposure to LDPP. Milk yield steadily decreased over the duration of the experiment in both light treatment groups. However, the extent of the decrease in milk yield was less in those animals exposed to LDPP. These findings are consistent with those of Russo et al. [6] who found that the effect of LDPP on milk yield was greater in late than in early lactation. The study by Garcia-Hernandez, et al. [3] conducted in the Northern Hemisphere found that average milk yield increased by 15% in goats exposed to LDPP during the final 6 months of lactation but the response was greatest over the last 3 months of lactation. On the other hand, the increase in milk yield in response to LDPP declined over the final 3 weeks in subtropical goats kidding in late Autumn and exposed to LDPP (14 h) for the first 110 days of lactation [11]. 

At least part of the difference between milk yields of the two light treatment groups may be due to the persistence of lactation in the LDPP group. Of the 61 goats that were exposed to the natural light, 8 dried off prior to the completion of the experiment, whilst none of the 61 exposed to LDPP dried off. There is a negative relationship between production and persistence with dairy cows producing higher yields drying off earlier [12]. It appears that light exposure can over-ride the negative relationship between persistency and production in lactating dairy goats. The present data demonstrate that light treatment is highly effective in increasing milk yield during autumn and winter in mid to late lactation goats confirming our previous observations [6].

Goat breeds are affected differently by photoperiod as seen in Alpine goats [13] and Creole goats [14] exposed to tropical conditions. The present study demonstrated that under the natural Southern Hemisphere winter photoperiod lactating goats maintain their elevated ovulatory activity. These findings are consistent with observations in Creole goats at a similar latitude in Argentina, though those goats were not lactating [15]. Similarly, a peak in oestrous activity (oestrus and ovulation) of goats was observed around the winter solstice [16]. Ovulatory activity increased gradually from February to April remaining elevated to July then gradually declining from August to September in the Southern Hemisphere [16]. Ovulatory activity declined significantly from June to August in the LDPP group indicating the breeding season was shortened by the light treatment. 

The ovulatory activity in the LDPP group appeared to be similar in those observed by others during spring [15]. The decline in ovulatory activity during LDPP is supported by Garcia-Hernandez, et al. [3] who looked at reproductive performance at parturition and found that a higher proportion of LDPP goats were pseudo-pregnant compared to goats under natural light conditions which led them to speculate that LDPP was detrimental to reproductive performance. On the other hand, LDPP exposure in goats in the sub-tropics did not decrease ovulation rate in another study [11]. Rivera et al. [15] found that reproductive activity late in the breeding season had a higher proportion of detached cycles where a silent ovulation occurred, or oestrous behaviour only was observed. It is possible that this may have been a factor that cannot be discounted in the present study especially in the LDPP group. Slight differences between the present results and previous studies investigating the effect of season on ovulatory activity may be attributed to the commercial setting in which the present study was conducted, and the photoperiod treatment applied. For example, environmental stimuli, including photoperiod, availability of food and social interactions [16,17] should be considered as potential regulators of seasonal reproduction. 

The difference in progesterone concentration between the light treatments are consistent with previous work where higher plasma progesterone concentrations and ovulatory activity were observed in goats induced to ovulate during the breeding season compared to those goats induced during the non-breeding season [18]. The shift in photoperiod in the current experiment presumably simulates the natural difference during the breeding and non-breeding season. The lower plasma progesterone concentrations in the LDPP goats as the study progressed may be associated with irregular cycles that are more frequently observed during the late breeding season. In this context, irregular cycles (either oestrus only or silent ovulation) were more frequently observed either early or late rather than during the peak of the breeding season [15]. 

In the present study the buck effect was shown to override the effect of LDPP on ovulation, but only during the month of August when ovulatory activity was already low. In contrast, buck exposure had no effect on oestrus or ovulation in goats housed in the sub-tropics although in that study there were high rates of oestrus (100%) and ovulation (95%) in the does exposed to LDPP [11]. The present experiment was conducted in a commercial setting with does that were exposed to bucks being reintroduced into the group. The presence of buck olfactory cues and their affect on does cannot be excluded and could possibly affect non buck exposed does. A study by Restall et al. [19] found that oestrous does could induce oestrus in anovulatory does. The presence of ovulatory does may have affected the results of this experiment. In the present study, males were kept separate from the milking herd though it cannot be assumed that they were far enough away that does could not detect buck audible and olfactory signals. 

Plasma prolactin concentrations were increased during LDPP with the effect being apparent at the earliest sampling time (ca. 2 months) and maintained for at least 4 months. The increase in the concentration of prolactin was an expected response as it has previously been observed in dairy ruminants including goats [3,6,20,21]. While it has generally been thought that the increase in prolactin secretion is not the cause of the effect of LDPP on milk production, the increase in plasma prolactin concentrations are evidence of an endocrinological response to LDPP [22,23]. However, recent data suggests that there may be a galactopoietic role for prolactin in ruminants since dopamine agonists decrease prolactin secretion and milk production in goats and dairy cows whereas the converse is true for dopamine antagonists [24]. Therefore, it is possible that the effect of LDPP on milk production is at least partially mediated via increased prolactin secretion. Interestingly, plasma prolactin concentrations also increased with time in the control goats as daylength increased after the winter solstice (21 June), although they were still markedly lower than in does exposed to LDPP. The increase in plasma prolactin in the control does after the solstice is probably insufficient to mitigate the against normal decline in milk yield as lactation advances.

## 5. Conclusions

This study is the first conducted in a commercial setting to investigate the effects of extended light treatment on reproduction and possible interaction between buck effect, using progesterone concentration as an indicator of ovulation. Milk yield was found to correspond to previous studies where extended light was observed to increase milk yield. Lactation persisted longer and produced a higher yield under extended lighting. The increase in prolactin concentrations were observed in the LDPP group and was used as an indicator of the effect of light exposure. The increase in plasma prolactin concentrations supports the hormonal changes found in goats under natural fluctuations of photoperiod over the year. This study had large numbers of treated and control goats that give the results validity. The results of this study indicate that extended light treatment could successfully be applied on a commercial scale to increase milk yield of dairy goats in Australia though it may be detrimental to reproductive performance.
